# Supplementary material for: Supraclavicular versus infraclavicular approach in inserting totally implantable central venous access for cancer therapy: A comparative retrospective study
Source: PLoS One. 2020 Nov 24;15(11):e0242727. doi: 10.1371/journal.pone.0242727 (PMC7685501; doi:10.1371/journal.pone.0242727)
Supplement: Attachment — Submitted filename: PLOS one comments .docx [file pone.0242727.s002.docx]

Dear editors,

We would like to thank you for your feedback and inform you that all comments were followed.

Responses to reviewer comments :

**Comment 1 :**  Please explain whether the Supraclavicular and infraclavicular approach for inserting totally implantable central venous access for cancer therapy considered standard care at your institution, or whether the procedures were being evaluated for research purposes.

Response : Indeed both supraclavicular and infraclavicular approaches in inserting the totally implantable central venous access are adopted as standard procedure by our institution and surgeons are trained on mastering both techniques. We completed the “Implantable ports and access to central vein’ paragraph in the methods section with a sentence making that precision in response to your commentary.

**Comment 2** : Please provide more information about what criteria used to determine which patients received which procedure and whether this decision was made as part of routine clinical care.

Response : The choice of which patient received which procedure was based on the period in which they underwent the operation. For the first half of the study period all patients systematically received an infraclavicular approach while the patients in the second half underwent the central venous access implantation by the supraclavicular approach. The required addition to the methodology was made.
