# Supplementary material for: Supraclavicular versus infraclavicular approach in inserting totally implantable central venous access for cancer therapy: A comparative retrospective study
Source: PLoS One. 2020 Nov 24;15(11):e0242727. doi: 10.1371/journal.pone.0242727 (PMC7685501; doi:10.1371/journal.pone.0242727)
Supplement: Attachment — Submitted filename: Yoffa comments.docx [file pone.0242727.s003.docx]

Dear editors

We would like to thank you for your feedback and inform you that all comments were followed.

Firstly, we would like to inform you that we are submitting our minimal underlying data set as an additional supplementary file according to PLOS journals requirement. All potentially identifiable patient information was fully anonymized.

Secondly, the responses to reviewer comments are the following:

Reviewer #1:

**1- Please mention the type of the study method in the title.**

The required change has been made.

**2- Introduction: The hypothesis and objective of the study should be explained in detail in the introduction part.**

The required change has been made.

**3- Methods: The sample size may not be sufficient to determine rarely seen early and late complications.**

Indeed the sample size is a limitation of our study which was mentioned in our limitations paragraph in the discussion. However, a future study with a bigger sample of patients should take place.

**4- In the sentence: “Group A patients received TIVAD through a supraclavicular approach and group B through an infraclavicular approach in the first and second half of our study period respectively (see details below).” Which details below where? A table? Insertion techniques?**

The mention refers to the Table 1 in the results section and was rectified.

**5- Results : Duration of catheter placement was mentioned in discussion part but there is no data in the results.**

The duration of catheter placement was already mentioned in paragraph 2 from the results section as well as the results section, Table 2, under the Mean operative time.

**6- Discussion : Personally, I do not agree with the authors about the presumption that ultrasound guided catheter placement may lead to deskilling. Although this is arguable, I believe that us guided procedures increase anatomically perception of the performers regarding the anatomic structures as they see them every time they perform the procedure (see anatomic variations as well).**

This section was modified.

Reviewer #2:

**1- Abstract: "yet the supraclavicular technique has been underused and never compared to the other method". No true - you yourselves reference several published comparisons!**

indeed the different approaches in central line placement have been previously discussed, however, this is the first comparison of both techniques in port catheter implantation.

**2- Abstract: (1,4% vs 7% p=0,21). This is a large difference without significance. Ranges should be included with the data to understand why this is so. In the results table the figure is 1.4 vs 1.5. Typo?**

This was a typo which we rectified.

**3- The text needs clarity. The techniques you are talking about need to be made very clear in the introduction, as at the moment it is a little confusing for me as a reader. For example: Be clear in the abstract and the introduction that you are just assessing access to the subclavian vein.**

**"Accordingly, the most frequent central venous catheterization approaches vary between the subclavian which is often adopted during surgical procedures and the internal jugular used for resuscitation. The use of the supraclavicular approach on the other hand is less usual. In fact, the supraclavicular technique for central vein puncture was first described In 1965 by Yoffa [2]."**

**The phrasing of this paragraph makes it seem like you are discussing subclavian access to supraclavicular IJV access, due to structure. If unfamiliar with Yoffa's technique (which I was before reading your paper, so thank you for the opportunity to improve my knowledge on the topic), then it is unclear that your study is actually comparing the supra and infra clavicular approach to subclavian vein access alone. This could be clarified with a short section describing the two techniques you are comparing clearly. The Plos-one readership is generalist.**

The required change has been made to the abstract and introduction.

**4- Methods: The time period for collection is short - By your results, the volume of cases in your centre is high. The numbers you have included exceed those in other literature. However your analysis would be even more powerful with greater numbers.**

We agree that a bigger sample would benefit and support this technique better. This retrospective study aimed to present a preliminary scientific basis for future randomized trials since no previous paper describes this approach for catheter implantation.

**5- Methods: The study is not randomised. This is a major limitation, and could be resolved easily. I base this on the methodology stating the team used one technique first for a month, then simply switched over. Why not randomised?**

We agree with your opinion, however, it is difficult to receive ethical approval for a randomized trial on a technique which was never presented in a scientific publication. As such, we reported this series to present this approach for the first time in literature.

**6- Methods: . The follow up for secondary outcomes is over a year earlier than the study period. Is this a typo?**

This was a typo which we rectified.

**7- Results: You start by using the term subclavicular, whereas you've used infraclavicular throughout the text.**

The required change has been made and the same term used throughout the manuscript.

**8- Discussion: This statement is not accurate - reference 17 specifically identifies benefits of US guidance for subclavian access from a recent meta-analysis, although not significant. "US guidance is associated with frequent risk of posterior vessel wall penetration as well as more lateral puncture sites as in the landmark puncture technique which may lead to pleural injury."**

This statement was removed from the manuscript.

**9- Minor comments : Abstract: ICA is used without prior reference to the phrase it represents ;**

We referenced the abbreviation as requested.

**10- The complications being studied are the primary outcome (I assume?). Please include information in the abstract.**

We specified our primary outcomes in the abstract as requested.

**11- Author needs to confirm - was the jugular approach also performed without US?**

We would like to confirm that the jugular approach was also performed without US

**12- Some minor spelling mistakes**

The manuscript grammar and spelling were revised.
