# Supplementary material for: Supraclavicular versus infraclavicular approach in inserting totally implantable central venous access for cancer therapy: A comparative retrospective study
Source: PLoS One. 2020 Nov 24;15(11):e0242727. doi: 10.1371/journal.pone.0242727 (PMC7685501; doi:10.1371/journal.pone.0242727)
Supplement: Attachment — Submitted filename: Response to comments ; yoffa.docx [file pone.0242727.s004.docx]

Dear editors,

Firstly, we would like to thank you for your feedback and constructive criticism. As requested, we provided a shorter title for our manuscript.

As regards the reviewer comment : **“ Reviewer #1: The authors are appreciated for their effort however, the required revisions were insufficiently done. Non-randomized characteristics of the study is a flaw. Ultrasound guidance is a standard for central venous access which is considered mandatory for increasing procedural success and reducing complications. Larger sample size is needed for assessing rare early and late complications “**

As previously mentioned, we did acknowledge the limitation originating from the non randomized character of the study in the limitations section as well as specified the need to consider our results with caution :  **“Limitations of this study are the retrospective aspect, absence of randomization and the fact that the results of this study were directly dependent on the senior surgeon's experience and skill. Therefore, caution is required when applying our results”**

We also acknowledge the sample size limitation, and the need for a larger sample in our future investigations regarding this subject. In fact, we believe this study provides us with guidance as we added to our limitation section. **“ An additional limitation is the relatively small number of patients included, however, this will allow us to decide the required sample size for future studies addressing this technique.”**

On the other hand, we agree that ultrasound guidance is the standard of use for central venous access as mentioned in our conclusion. However, we made modifications to our conclusion to further emphasize on ultrasound guidance prioritization. In fact, central line placement is a procedure conducted by radiologists, intensive care specialists and surgeons with the latter using landmark techniques more frequently. This is particularly more frequent in low and middle income contexts where the availability of ultrasounds devices is not always possible, due to resource restrictions. In this case and only in the absence of ultrasound guidance is the yoffa approach preferred to other techniques.
