# Supplementary material for: Supraclavicular versus infraclavicular approach in inserting totally implantable central venous access for cancer therapy: A comparative retrospective study
Source: PLoS One. 2020 Nov 24;15(11):e0242727. doi: 10.1371/journal.pone.0242727 (PMC7685501; doi:10.1371/journal.pone.0242727)
Supplement: Attachment — Submitted filename: Comments to the authors.docx [file pone.0242727.s005.docx]

Comments to the authors

I read the article of the authors titled "Supraclavicular versus infraclavicular approach in inserting totally implantable central venous access for cancer therapy : a comparative retrospective study" with interest. When I examined the answers given to the previous criticisms, I saw that the necessary corrections were made. However, I think I wish the manuscript had been sent to those who made the initial referee evaluation. Because, as a port catheter surgery practitioner, I have to ask many different questions from the publication. If I ask questions again, it would be unfair to both the authors and the publisher. Although the article seems suitable for the publication in terms of its technique and subject, the final decision of the valuable editor will be decisive.

yours sincerely
